# Thermally Conductive Polydimethylsiloxane-Based Composite with Vertically Aligned Hexagonal Boron Nitride

**DOI:** 10.3390/polym16223126

**Published:** 2024-11-08

**Authors:** Haosen Lin, Genghao Xu, Zihao Chen, Luyang Wang, Zhichun Liu, Lei Ma

**Affiliations:** 1 College of New Materials and New Energies, Shenzhen Technology University, Shenzhen 518118, China; 2210412045@stumail.sztu.edu.cn (H.L.); 2210412038@stumail.sztu.edu.cn (G.X.); 2310412093@stumail.sztu.edu.cn (Z.C.); wangluyang@sztu.edu.cn (L.W.); 2 School of Energy and Power Engineering, Huazhong University of Science and Technology (HUST), Wuhan 430074, China; zcliu@hust.edu.cn

**Keywords:** boron nitride, thermal interface material, high thermal conductivity, vertical alignment

## Abstract

The considerable heat generated in electronic devices, resulting from their high-power consumption and dense component integration, underscores the importance of developing effective thermal interface materials. While composite materials are ideal for this application, the random distribution of filling materials leads to numerous interfaces, limiting improvements in thermal transfer capabilities. An effective method to improve the thermal conductivity of composites is the alignment of anisotropic fillers, such as hexagonal boron nitride (BN). In this study, the repeat blade coating method was employed to horizontally align BN within a polydimethylsiloxane (PDMS) matrix, followed by flipping and cutting to prepare BN/PDMS composites with vertically aligned BN (V-BP). The V-BP composite with 30 wt.% BN exhibited an enhanced out-of-plane thermal conductivity of up to 1.24 W/mK. Compared to the PDMS, the V-BP composite exhibited outstanding heat dissipation capacities. In addition, its low density and exceptional electrical insulation properties showcase its potential for being used in electronic devices. The impact of coating velocity on the performance of the composites was further studied through computational fluid dynamics simulation. The results showed that increasing the coating velocity enhanced the out-of-plane thermal conductivity of the V-BP composite by approximately 40% compared to those prepared at slower coating velocities. This study provides a promising approach for producing thermal interface materials on a large scale to effectively dissipate the accumulated heat in densely integrated electronic devices.

## 1. Introduction

Recently, the demand for integration and power density in electronic devices has been growing rapidly, driven by the development of transformative technologies like 5G communications and artificial intelligence [1,2]. This rapid increase in power use and component integration leads to considerable heat generation and accumulation within these devices, which severely compromises their reliability and lifespan [3]. Thermal interface materials (TIMs) are used as a filling layer between electronic devices and heat sinks and play a crucial role in thermal management.

Polymer-based TIMs have been extensively investigated due to their high cost efficiency, effective electrical insulation, and high flexibility [4,5]. Although the very high thermal conductivity of intrinsic TIMs can be achieved by aligning polymer chains, the complex preparation process limits their large-scale production and application [6,7,8]. Hence, the integration of highly thermally conductive materials, such as carbon-based materials [9,10,11,12,13,14], metal and alloys [15,16,17], and ceramics [18,19,20,21], into polymers to improve thermal transfer capabilities has attracted significant attention.

The TIMs used in electronic devices require electrical insulation alongside thermal conductivity. Therefore, the development of thermally conducting yet electrically insulating TIM composites is of paramount importance. Hexagonal boron nitride (BN), often referred to as white graphene due to its graphite-like layered crystal structure, is a two-dimensional material characterized by its high in-plane thermal conductivity and exceptional electrical insulation properties, making it a promising candidate for heat dissipation in electrical and electronic fields [22,23]. However, simply incorporating BN into polymers offers limited enhancement in thermal conductivity and often inhibits mechanical performance due to the introduction of numerous interfaces [24]. The key to fully harnessing the in-plane thermal transfer capabilities of BN without compromising the mechanical properties of the final composite lies in the well-organized alignment and the continuous thermal pathways within the material [25], and several strategies have been investigated. Chen et al. [18] successfully fabricated a BN-based skeleton using a salt-template method, followed by impregnation with epoxy, achieving a composite with a λ of 1.227 W/mK. Sun et al. [26] created a boron nitride nanosheet/poly-p-phenylene benzodiazole composite film by self-assembling one- and two-dimensional materials, leading to an impressive $\lambda_{∥}$ of 45.15 W/mK. He et al. [27] engineered locally graded orientations of BN within composites using an external magnetic field, resulting in a λ of up to 12.1 W/mK along the alignment direction. While these strategies demonstrate the potential of aligned BN fillers, they involve complex processing techniques. In contrast, the shear-assisted alignment method [28,29,30,31,32,33,34] is a simpler and more effective way to align fillers within composites along the shearing direction. Kong at al. [35] proposed a multi-casting strategy to prepare BN/polydimethylsiloxane (PDMS) composites and studied the impact of casting thickness on their thermal conductivity. However, a systematic analysis on the impact of the coating velocity on the orientation of BN in the composites is still lacking.

In this study, the repeat blade coating strategy was employed to prepare BN/PDMS composites with horizontally aligned BN (H-BP) to the desired thickness, followed by cutting and flipping to prepare the composites with vertically aligned BN (V-BP). The repeat blade coating strategy could effectively improve the thermal conductivity of the V-BP composites by aligning the BN to establish pathways for heat transfer, resulting in an enhanced $\lambda_{⊥}$ of 1.24 W/mK at only a 30 wt.% BN loading. Additionally, the impact of the coating velocity on the thermal conductivity of the composites was also investigated using computational fluid dynamics simulation, indicating that the thermal conductivity of the composites increased with the coating velocity due to the growing shearing effect.

## 2. Materials and Methods

### 2.1. Materials

Polydimethylsiloxane 184 silicone elastomer (components A and B) was purchased from Dow Chemical Co., Ltd., Midland, MI, USA. Hexagonal boron nitride (BN) with an average lateral size of 5–10 μm was provided by Beijing MREDA Technology Co., Ltd., Beijing, China. All the materials were used without any purification.

### 2.2. Preparation of BN/PDMS Composites

In this study, the sample names are designated according to V/H-BP-x-y, unless otherwise stated. V/H corresponds to a BN/PDMS composite with vertically/horizontally aligned BN, while x and y represent the loading of BN (wt.%) and the coating velocity (mm/s), respectively. R-BP-x corresponds to the BN/PDMS composite with an x wt.% loading of randomly aligned BN.

**Figure 1:**
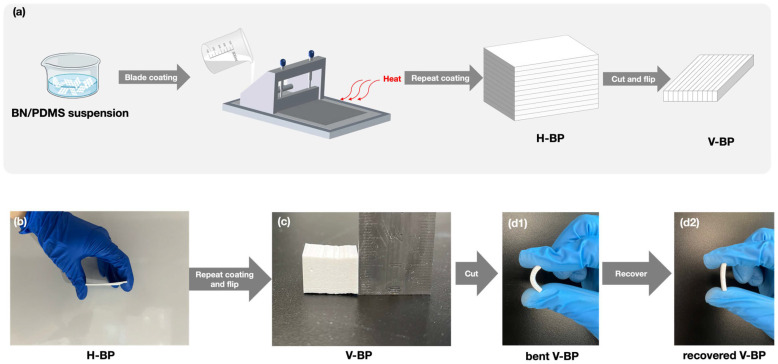
(**a**) Schematic illustration of V-BP composite preparation. Optical images of (**b**) H-BP-30-100 composite, (**c**) V-BP-30-100 composite, and (**d1**,**d2**) bent and recovered V-BP-30-100 composite, respectively.

Figure 1a illustrates the preparation of the BN/PDMS composite with vertically aligned BN (V-BP). First, the PDMS components A and B were mixed in a 10:1 weight ratio using a planetary vacuum centrifugation agitator. The appropriate amount of BN was then added, and then was further blended to homogeneity using a planetary vacuum centrifugation agitator followed by an overhead agitator. The blade coating thickness was fixed at 100 μm, and the coating velocity was controlled by adjusting the motor power. The BN/PDMS suspension was then cured in situ using heating lamps to form the BN/PDMS composite with horizontally aligned BN (H-BP) (Figure 1b). The composite was cooled to room temperature with a semiconductor refrigerator after it was cured. Then, the blade thickness was increased by 100 μm, and the next layer was coated on top of the previous layer. This process was repeated until the desired thickness was achieved. Finally, the H-BP composite was flipped and cut to prepare the V-BP composite (Figure 1c).

The BN/PDMS composite with randomly distributed BN (R-BP) was prepared by pouring the BN/PDMS suspension into a polytetrafluoroethylene mold, followed by curing it in the vacuum dryer at 80 °C for 4 h.

### 2.3. Characterization

A field emission scanning electron microscope (FE-SEM, Gemini 300, Carl Zeiss Microscopy Ltd., Cambridge, UK) operating at 15 kV was used to observe the microstructures. Powder X-ray diffraction (XRD) patterns were acquired using an X-ray diffractometer (SmartLab XRD, Rigaku, Tokyo, Japan) at a scanning range (2θ) from 20° to 50° and rate of 10°/min. The specific heat capacity was measured using a differential scanning calorimeter (DSC 3, METTLER TOLEDO, Greifensee, Switzerland) under an N_2_ atmosphere. Thermal diffusivity was measured three times for each material with a laser flash machine (LFA-467, NETZSCH, Selb, Germany) at 25 °C. An infrared camera (Ti 480 Pro, FLUKE, Everett, WA, USA) was used to record the thermal diffusion process. The density of the composites was measured based on the Archimedes principle using absolute ethyl alcohol as a measurement liquid. The universal tensile tester (ZQ-950B, Zhiqu Precision Instruments Co., Ltd., Dongguan, China) was used to test the mechanical properties of the composites. The apparent viscosity of the BN/PDMS suspension was analyzed in the shear rate range of 0.1 s^−1^ to 100 s^−1^ using a rheometer (MCR 102e, Anton Paar, Graz, Austria) equipped with a parallel plate (diameter of 25 mm). Computational fluid dynamics (CFD) simulation was performed using Ansys CFD 2022.

## 3. Results

### 3.1. The Preparation of the V-BP Composites

The V-BP composites were fabricated by flipping and cutting the H-BP composites, which were prepared through the repeat blade coating strategy, as shown in Figure 1a. Figure 1(d1,d2) show that the prepared V-BP composite is resilient and retains its shape after bending, indicating that the outstanding flexibility of the PDMS matrix was maintained due to the well-aligned BN skeleton. The simple preparation method allows for large-scale production of the V-BP composite and can be extended to fabricate composites with other anisotropic filling materials that have exceptional thermal transfer capabilities.

### 3.2. The Thermal Conductivity and Mechanical Properties of the V-BP Composite

**Figure 2:**
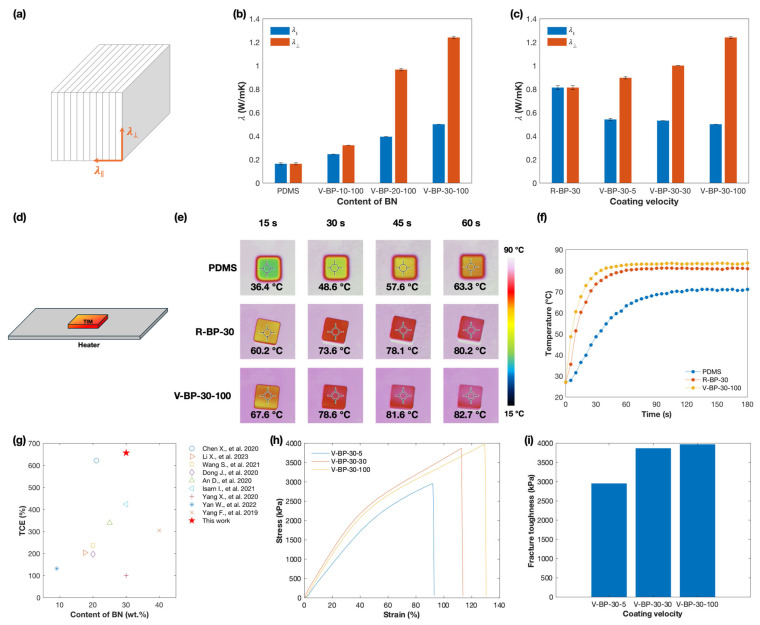
(**a**) Direction of $\lambda_{⊥}$ and $\lambda_{∥}$. (**b**) Thermal conductivity of the V-BP composite with different contents of BN prepared at a coating velocity of 100 mm/s. (**c**) Thermal conductivity of V-BP-30 composites prepared at different coating velocities. (**d**) Illustration of the performance test for thermal diffusion. (**e**) IR camera images of the surface of the composites and (**f**) the temperature variation in the composites while heating. (**g**) Comparison of the thermal conductivity enhancement of the V-BP-30-100 composite with BN-containing composites reported in References [18,37,38,39,40,41,42,43,44]. (**h**) Tensile stress–strain curve and (**i**) fracture toughness of the V-BP composites with a 30 wt.% loading of BN.

The thermal conductivity (W/mK) of the material was calculated by

(1) $$\lambda =a\times \rho \times C_{p}$$

where a, ρ, and $C_{p}$ refer to the thermal diffusivity (m^2^/s), density (kg/m^3^), and specific heat capacity (J/(kg⋅K)), respectively. Figure 2a demonstrates the direction of $\lambda_{∥}$ and $\lambda_{⊥}$, which are perpendicular and parallel to the coating plane, respectively. As shown in Figure 2b, both $\lambda_{∥}$ and $\lambda_{⊥}$ increase with the loading of BN from 0 to 30 wt.%, indicating the formation of more continuous thermal transfer pathways with an increased BN content. Additionally, the influence of the coating velocity on the thermal conductivity was studied. Figure 2c shows that the $\lambda_{⊥}$ of the V-BP-30 composite increases with the coating velocity, indicating that the faster coating velocity involves more powerful shearing forces, which increases the alignment of BN. Specifically, the $\lambda_{⊥}$ of the V-BP-30-100 composite reaches a value of 1.24 W/mK. This result is an improvement of 675% and 53% compared to the PDMS matrix and the R-BP-30 composite, respectively, and is 38% higher than the $\lambda_{⊥}$ of V-BP-30-5. Theoretically, the $\lambda_{⊥}$ of the V-BP composites should continue increasing with the coating velocity, reaching a peak when all the hexagonal boron nitrides are fully aligned. However, the high viscosity of the BN/PDMS suspension constrains this process. During blade coating, the suspension requires time to flow into the gap between the blade and the base, as shown in Figure S5(a,b), to enable smooth coating on the base. At high coating speeds, the suspension tends to adhere to the blade rather than coating on the base effectively. The $\lambda_{∥}$ of the V-BP composites is lower than the corresponding $\lambda_{⊥}$ and decreases as the coating velocity increases. This trend indicates an increase in the anisotropy of the composites, which is attributed to the more organized alignment of the sheet-like BN (Figure S1). Figure 2g summarizes the thermal conductivity enhancement (TCE) of BN-containing composites reported in other studies and compares it with that of the V-BP-30-100 composite. The TCE [36] represents the increase in thermal conductivity over the polymer matrix, which is calculated by

(2) $$TCE=\frac{\lambda_{C}-\lambda_{P}}{\lambda_{P}}\times 100\%$$

where $\lambda_{C}$ and $\lambda_{P}$ are the thermal conductivity (W/mK) of the composite and the polymer matrix, respectively. It is evident that the V-BP-30-100 composite exhibits a comparable thermal conductivity enhancement.

The thermal transfer performances of the PDMS matrix, the R-BP-30 composite, and the V-BP-30-100 composite were evaluated under the same heating conditions. All the samples were shaped with dimensions of 10 × 10 × 3 cm and placed on the heater, as shown in Figure 2d. A commercial infrared camera was used to record the surface temperature. As shown in Figure 2e,f, the heat transfer speed of the V-BP-30-100 composite was the fastest among the three samples, reaching to its peak temperature of 83.0 °C in 70 s, while the PDMS matrix and the R-BP-30 composite could only reach up to 71.1 °C in 135 s and 81.1 °C in 90 s, respectively. This result shows that the V-BP-30-100 composite can reach the highest saturation temperature in the shortest time. In conjunction with its low density (Figure S4) and exceptional electrical insulation between the BN and PDMS matrix, these properties exhibit the potential application of this composite material in the effective thermal management of electronic devices.

Figure 2h,i show that the elongation at break and fracture toughness of the V-BP composite with a 30 wt.% BN loading both increase with the coating velocity, indicating improved flexibility and anti-fatigue performance. With an increasing coating velocity, the orientation of BN improves, effectively reducing the stress concentration in the composite.

### 3.3. The Impact of Coating Velocity on the Orientation of BN

**Figure 3:**
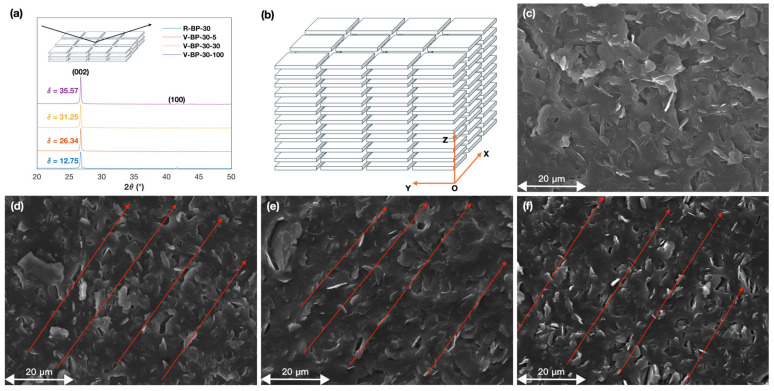
Analysis of the orientation of BN in the composite. (**a**) The XRD pattern of V-BP-30 composites prepared at different coating velocities and the R-BP-30 composite. (**b**) Illustration of the ideal distribution of BN in the V-BP composite. The SEM images of (**c**) the R-BP-30 composite, (**d**) the V-BP-30-5 composite, (**e**) the V-BP-30-30 composite, and (**f**) the V-BP-30-100 composite.

The outstanding thermal transfer capabilities of the V-BP-30-100 composite compared to the PDMS matrix and to the R-BP-30 composite are rooted in the vertical alignment of BN. The XRD patterns of the R-BP-30 composite and the V-BP-30 composites prepared with different coating velocities were analyzed to investigate the orientation of BN in the materials. The characterization peaks at 26.9° and 41.7° corresponding to the (002) and (100) lattice planes of BN are shown in Figure 3a. The orientation degree of BN in the composites can be evaluated through the ratio of the intensity of these two characteristic peaks. The intensity ratio $I_{\left(002\right)}/I_{\left(100\right)}$ of the cross section of the V-BP-30-5 composite is 26.34, which is higher than that of the R-BP-30 composite. Additionally, the intensity ratios $I_{\left(002\right)}/I_{\left(100\right)}$ of the cross section of the V-BP-30 composites increase with the coating velocity, which reaches a maximum of 35.57 at a coating velocity of 100 mm/s. Also, the intensity ratios $I_{\left(002\right)}/I_{\left(100\right)}$ of the cross section of the V-BP composites with varied BN contents are greater than those of the R-BP composites, further indicating the well-ordered alignment of BN in the V-BP composites.

Figure 3b illustrates the ideal distribution of BN in the composites, where the coating direction is along the *Y* axis. The SEM images shown in Figure 3c–f correspond to the YOZ plane. The orientation of BN in these images is indicated by red arrows. Figure 3c shows that BN is randomly distributed in the R-BP-30 composite without any specific orientation. As shown in Figure 3d–f, BN is increasingly aligned along the direction parallel to the red arrows as the coating velocity increases, suggesting that blade coating can effectively align BN and the orientation of BN can be improved by a high coating velocity.

Additionally, the orientation of BN in the R-BP composites with various BN contents was compared to that of the corresponding H-BP composites. As shown in Figure S2a,d, the intensity ratios $I_{\left(002\right)}/I_{\left(100\right)}$ of the cross section of the V-BP-10-100 and the V-BP-20-100 composites were both significantly greater compared to the intensity ratios of the R-BP composites with the same BN loading. Also, Figure S2b,c,e,f show the SEM images demonstrating that the lateral sides of BN that could be observed in the V-BP-10-100 composite and the V-BP-20-100 composite are significantly more than those in the SEM images of the R-BP-10 composite and the R-BP-20 composite, respectively.

Based on the above analysis, the repeat blade coating strategy facilitates the alignment of BN in the composites, and the faster coating velocity is effective at inducing the orientation of BN, which improves the alignment of the heat channel and enhances the thermal transfer capabilities of the material.

### 3.4. Computational Fluid Dyanmics Simulation

To gain a comprehensive understanding of the influence of coating velocity on the alignment of BN, computational fluid dynamic (CFD) simulation was performed using the Ansys CFD 2022, and the BN/PDMS suspension with a 30 wt.% loading of BN was taken as the representative example. The shear rate $\dot{\gamma }$ and expansion rate $\dot{\varepsilon }$ were defined as ∂μ/∂y and ∂ν/∂y, respectively. In the formula, μ and ν are the velocity along the *x* and *y* axis, respectively.

Prior to the CFD simulation, the rheological properties of the BN/PDMS suspension with a 30 wt.% loading of BN were studied. Figure S3 shows that the suspension possesses shear thinning properties. The non-Newtonian fluid model Ostwald-De Waele power-law

(3) $$\mu =k\times {\dot{\gamma }}^{n-1}$$

was applied to fit the viscosity curve, where μ, $\dot{\gamma }$, k, and n are the dynamic viscosity, shearing rate, consistency index, and power-law index, respectively. The calculated result is in good agreement with the experimental data.

The velocity profiles along the coating direction u were calculated to evaluate their impact on the orientation of the horizontally aligned BN, and the results are shown in Figure S5(c1–c3). The maximum velocities equal to the coating velocities could be observed at the top surface of the BN/PDMS suspension. When the coating velocity is 100 mm/s, the maximum Reynolds number for the BN/PDMS suspension under a shearing rate of 100 s^−1^ is calculated according to

(4) $$Re=\frac{\rho \nu L}{\mu }$$

where ρ, ν, L, and μ are the fluid density, flow velocity, characteristic length, and dynamic viscosity, respectively. A Reynolds number smaller than 0.01 represents laminar flow, indicating that the horizontally aligned structures formed by the shearing force can be maintained [30,45,46].

**Figure 4:**
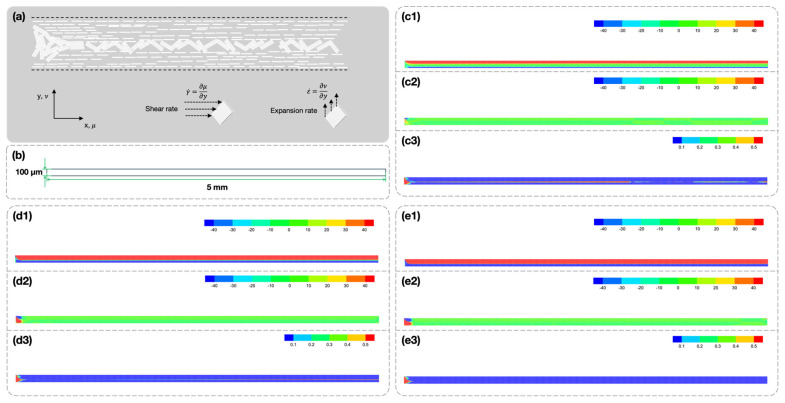
Impact of coating velocity on the horizontal alignment of BN. (**a**) Illustration of the rational processes of BN from random to parallel to the shearing direction. (**b**) Fluid model used in the Ansys CFD 2022 simulation. (**c1**–**e1**) The shearing rate, (**c2**–**e2**) the expansion rate, and (**c3**–**e3**) the absolute value of the ratio of the shearing rate and the expansion rate when the coating velocity is 5, 30, 100 mm/s, respectively.

Figure 4(c1–e1) show the shearing rate contours when the suspension was exposed to different coating velocities. The results show that the regions that were sheared expanded with increasing coating velocities. The expansion rate contours shown by Figure 4(c2–e2) have the same variation trends as the shearing rate contours. However, the large expansion rate can only be observed in the entrance section, while that of other regions is negligible, indicating that there is no significant negative impact on the horizontal alignment of BN in the suspension.

The absolute value of the ratio of the expansion rate and shearing rate, $\left|\dot{\varepsilon }/\dot{\gamma }\right|$, can be used to evaluate the alignment of BN [30,47]. A smaller $\left|\dot{\varepsilon }/\dot{\gamma }\right|$ ratio represents a better horizontal alignment of BN. In most of the regions of the model, a $\left|\dot{\varepsilon }/\dot{\gamma }\right|$ ratio smaller than 0.1 is observed when the coating velocity is 5 mm/s, as shown in Figure 4(c1), indicating that a large proportion of BN was aligned horizontally. However, a $\left|\dot{\varepsilon }/\dot{\gamma }\right|$ ratio greater than 0.5 is still present in a small region. This region decreases with an increase in the coating velocity. When the coating velocity is 100 mm/s, the region with a $\left|\dot{\varepsilon }/\dot{\gamma }\right|$ ratio greater than 0.1 is negligible except at the entrance section, indicating that the faster coating velocity is favorable for the horizontal alignment of BN.

Although the shearing rate and the expansion rate both increase with the coating velocity, the growth of the shearing rate is quicker than that of the expansion rate. As shown in Figure S6(b3,c3,d3), the maximum $\left|\dot{\varepsilon }/\dot{\gamma }\right|$ ratio decreases from over 1000 at a position 2 mm from the inlet with a coating velocity of 5 mm/s, to less than 0.5 at a position 5 mm from the inlet with a coating velocity of 100 mm/s. Additionally, the $\left|\dot{\varepsilon }/\dot{\gamma }\right|$ ratio at the entrance section remains large regardless of the coating velocity, but this region was cut and discarded after the curing of the BN/PDMS suspension. Hence, this region would not have any adverse impact on the thermal conductivity of the final V-BP composites.

### 3.5. Comparison of the Impact of the Coating Thickness and the Coating Velocity

In addition to coating velocity, coating thickness is another influential factor affecting the shearing rate [35]. In this work, the V-BP composites with a BN loading of 30 wt.% were prepared at a coating velocity of 100 mm/s and thicknesses ranging from 100 to 1000 μm. The sample names follow the format V-BP-x-y-z, where *x*, *y*, and *z* represent the BN loading (wt.%), coating thickness (μm), and coating velocity (mm/s), respectively. R-BP-x denotes BN/PDMS composites with a randomly aligned x wt.% loading of BN.

**Figure 5:**
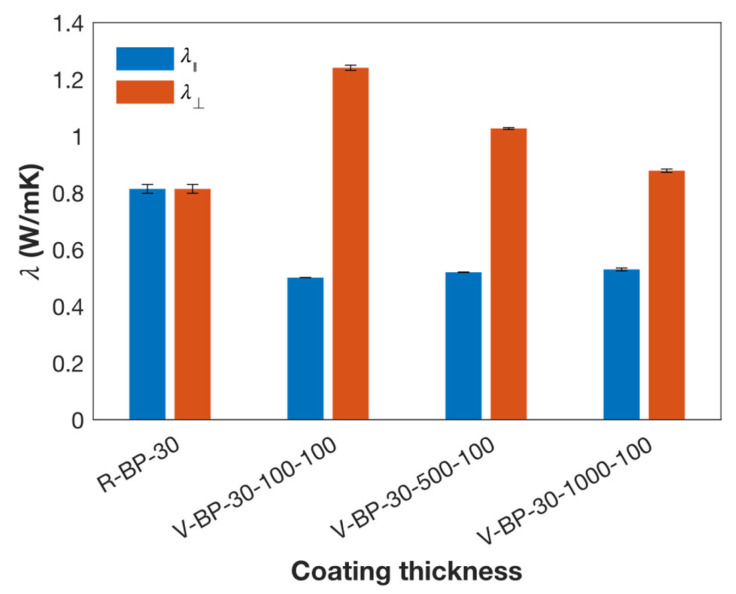
Thermal conductivity of V-BP-30 composites prepared at a coating velocity of 100 mm/s and different coating thicknesses.

Figure 5 shows that the $\lambda_{⊥}$ of the V-BP composites decreases as the coating thickness increases, consistent with previous findings. The $\lambda_{⊥}$ of the V-BP-30-100-100 composite is 41% higher than that of the V-BP-30-1000-100 composite. In this case, the coating thickness of the V-BP-30-100-100 composite is one-tenth of that of the V-BP-30-1000-100 composite, indicating that the shearing effect due to the coating thickness on the V-BP-30-100 composite is ten times stronger.

Regarding coating velocity, the $\lambda_{⊥}$ of the V-BP-30-100-100 composite is 38% higher than that of the V-BP-30-100-5 composite. The coating velocity for the V-BP-30-100-100 composite is twenty times that of the V-BP-30-100-5 composite, suggesting a twenty-fold increase in the shearing effect. Coating thickness exhibits more impact than coating velocity based on the result, while the V-BP composite prepared at a thinner coating thickness requires more time to cure and cool compared to those prepared at a thicker coating thickness and faster coating velocity, making it less suitable for production.

## 4. Conclusions

In summary, the simple repeat blade coating strategy was employed to fabricate PDMS-based composites containing vertically aligned BN. Under the shearing effect from repeat blade coating, a vertically oriented BN skeleton was obtained which is conducive to the formation of an effective heat channel. As a result, the $\lambda_{⊥}$ of the V-BP-30-100 composite was significantly higher than that of the PDMS matrix and the randomly aligned R-BP-30 composite, reaching up to 1.24 W/mK. The energy was transferred rapidly to the top surface of the V-BP-30-100 composite from the heated bottom surface. This effective thermal conductivity, in conjunction with its electrical insulation, demonstrates the potential of this composite in electronic thermal management. Additionally, the influence of the coating velocity on the orientation of BN in the composites was studied through CFD simulation. The ratio of the shearing rate and the expansion rate, which can be used to evaluate the alignment of BN, decreased with the faster coating velocity due to the larger growth rate of the shearing rate than that of the expansion rate. The $\lambda_{⊥}$ of the V-BP-30-100 composite increased by almost 40% compared to the V-BP-30-5 composite, indicating that the rapid coating velocity was favorable to the orientation of BN. These findings deliver key insights into the development of electrically insulating thermal interface materials.

## Data Availability

The original contributions presented in the study are included in the article and Supplementary Materials; further inquiries can be directed to the corresponding author.

## Supplementary Materials

The following supporting information can be downloaded at https://www.mdpi.com/article/10.3390/polym16223126/s1; Figure S1: SEM images of BN with an average lateral size of 5–10 μm; Figure S2: Analysis of the orientation of BN in the composites; Figure S3: Apparent viscosity of the BN/PDMS suspension with a 30 wt.% loading of BN; Figure S4: The solid density of the V-BP composites; Figure S5: Velocity along the coating direction (*x* axis); Figure S6: Detail of the simulation results. Table S1: Boundary setting of the CFD simulation; Table S2: Thermal conductivity of all composites prepared at a coating thickness of 100 μm; Table S3: Thermal conductivity of V-BP composites with a 30 wt.% loading of BN prepared at a coating velocity of 100 mm/s and different coating thicknesses ranging 100–1000 μm; Table S4: Thermal conductivity enhancement (TCE) of this work in comparison to other composites containing boron nitride reported by other studies. References [18,37,38,39,40,41,42,43,44] are included in the Supplementary Materials.
