# Supplementary material for: Thermally Conductive Polydimethylsiloxane-Based Composite with Vertically Aligned Hexagonal Boron Nitride
Source: Polymers (Basel). 2024 Nov 8;16(22):3126. doi: 10.3390/polym16223126 (PMC11598570; doi:10.3390/polym16223126)
Supplement: Supplementary file 1 [file polymers-16-03126-s001.zip › polymers-3275872-supplementary.pdf]

Supplementary materials for

Thermally Conductive Polydimethylsiloxane-Based Composite with Vertically Aligned Hexagonal Boron Nitride

Haosen Lin [1], Genghao Xu [1], Zihao Chen [1], Luyang Wang [1], Zhichun Liu [2] and Lei Ma [1,*]

[1] College of New Materials and New Energies, Shenzhen Technology University, Shenzhen 518118, China; 2210412045@stumail.sztu.edu.cn (H.L.); 2210412038@stumail.sztu.edu.cn (G.X.); 2310412093@stumail.sztu.edu.cn (Z.C.); wangluyang@sztu.edu.cn (L.W.)

[2] School of Energy and Power Engineering, Huazhong University of Science and Technology (HUST), Wuhan 430074, China; zcliu@hust.edu.cn (Z.L.)

* Correspondence: malei@sztu.edu.cn (L.M.)

Supplementary figures

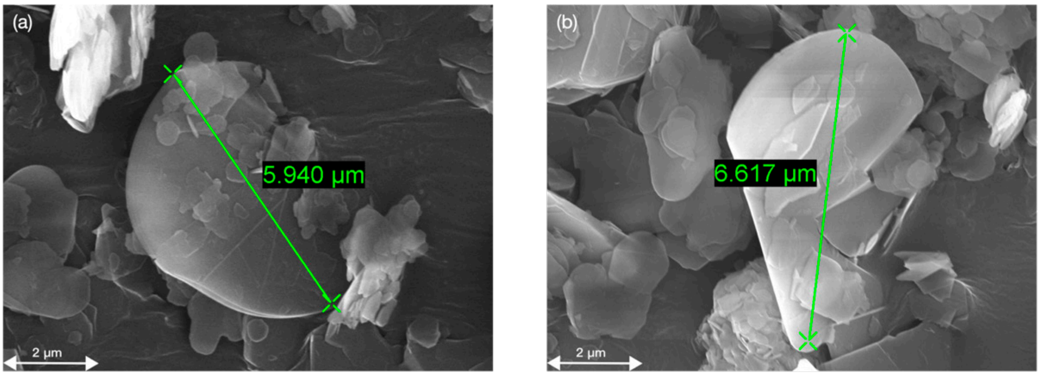

Figure S1 SEM images of BN with an average lateral size of 5–10 µm.

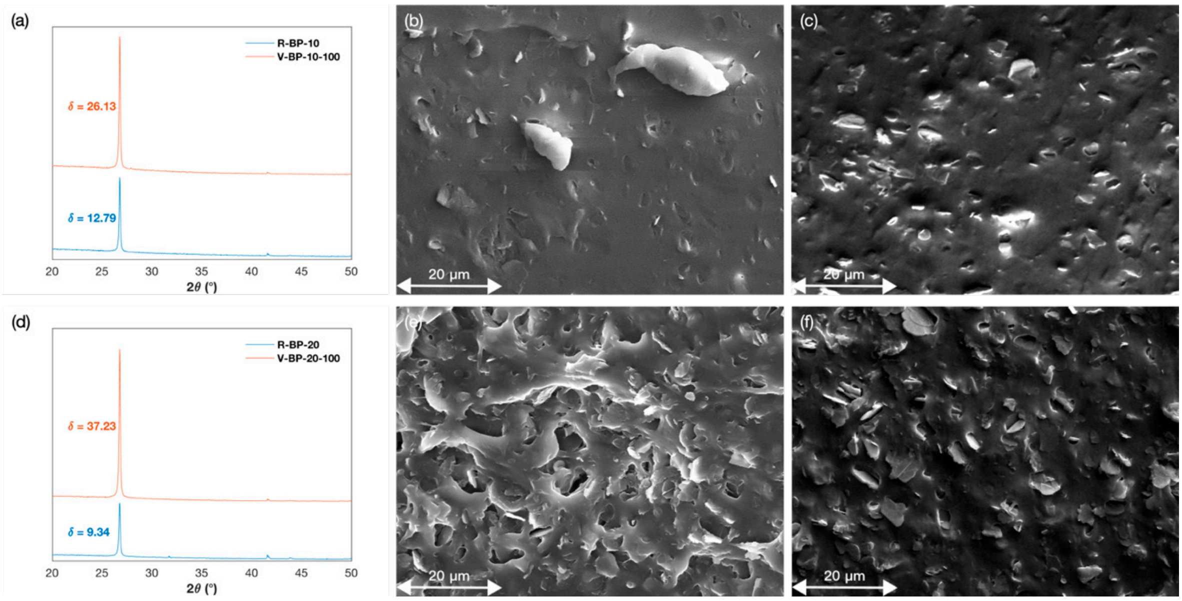

Figure S2 Analysis of the orientation of BN in the composite. XRD patterns of (**a**) the R-BP-10 composite and the V-BP-10-100 composite, and (**d**) the R-BP-20 composite and the V-BP-20-100 composite. The SEM images of (**b**) the R-BP-10 composite, (**c**) the V-BP-10-100 composite, (**e**) the R-BP-20 composite, and (**f**) the V-BP-20-100 composite.

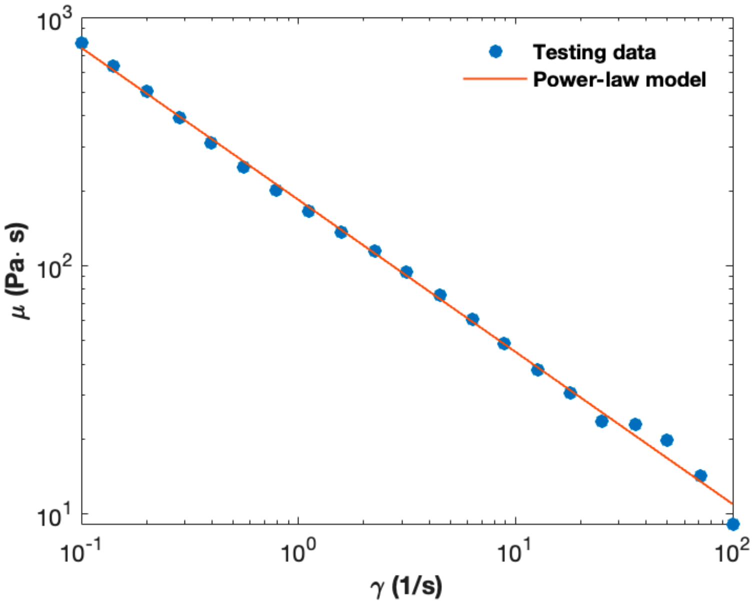

Figure S3 Apparent viscosity of the BN/PDMS suspension with a 30 wt.% loading of BN.

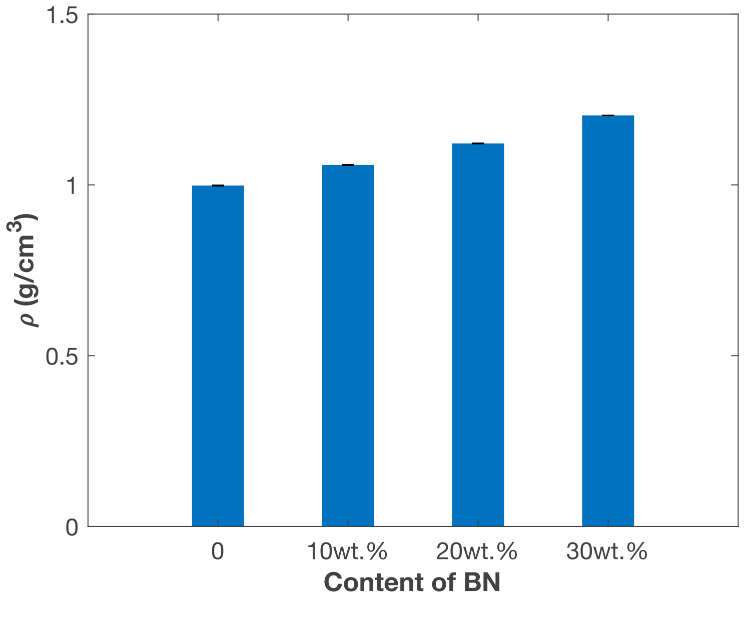

Figure S4 The solid density of the V-BP composites.

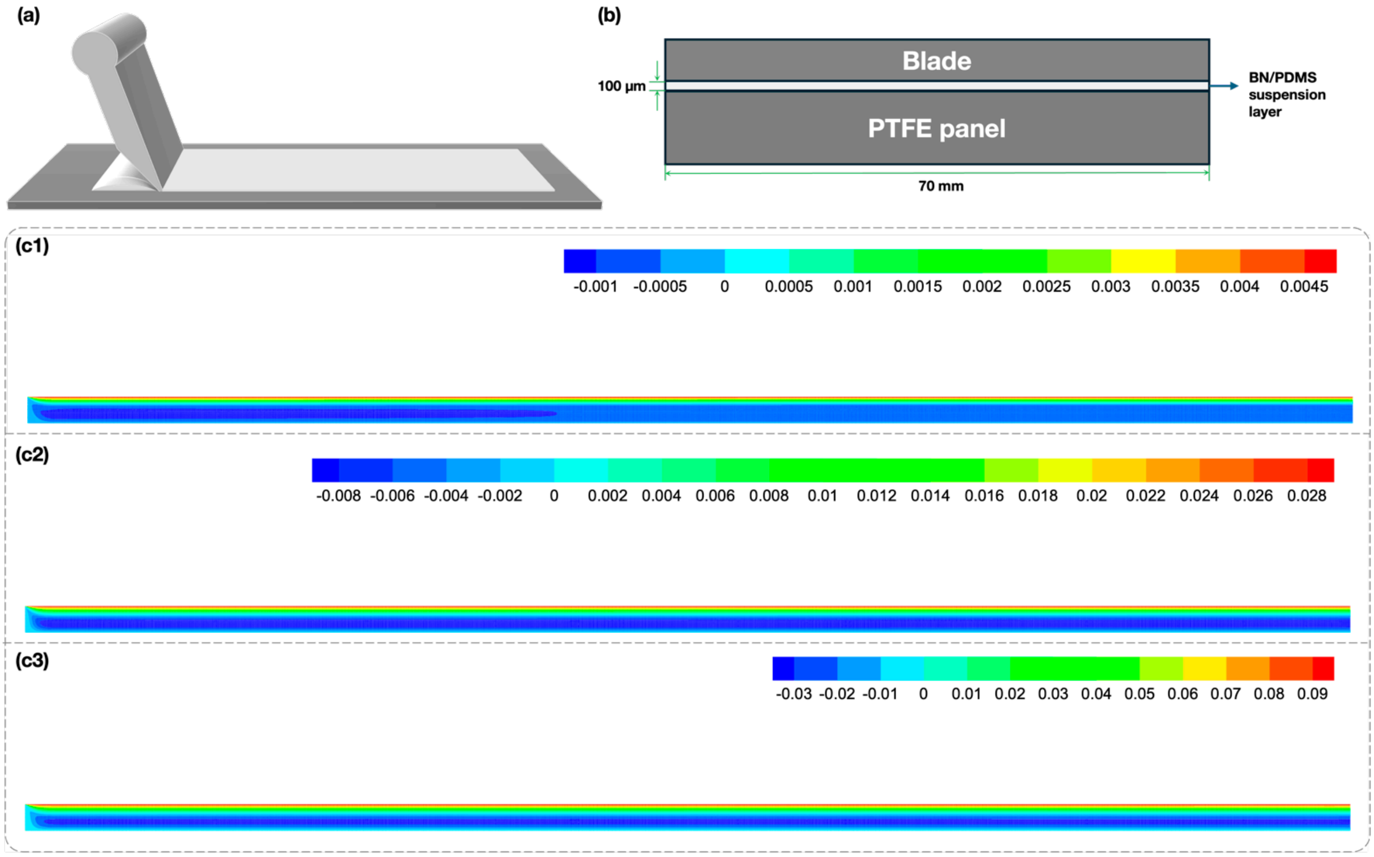

Figure S5 Velocity along the coating direction (x axis). (**a**, **b**) The front view and the side view of the blade coating preparation platform, respectively. (**c1**-**c3**) The velocity along the coating direction when the coating velocity is 5 mm/s, 30 mm/s, and 100 mm/s, respectively.

CFD simulation

The computational fluid dynamics (CFD) analysis was performed using the Ansys CFD 2022 to calculate the steady, single-phase and laminar flow in a rectangular fluid zone (5 mm × 100 μm) illustrated by Fig. S6 (a). The boundary settings are demonstrated by Table S1, the velocity of the Blade ranges from 0.005 m/s to 0.100 m/s. The apparent viscosity of the BN/PDMS suspension was analyzed in the shear rate range of 0.1 s^{-1} to 100 s^{-1} using a rheometer (MCR 102e, Anton Paar, Graz, Austria) equipped with a parallel plate (diameter of 25 mm), followed by the non-newton fluid model Ostwald-De Waele power-law $\mu = k \times \dot{\gamma}^{n-1}$ was employed to fit the viscosity curve. As shown by Fig. S3, the calculated result is in good agreement with the experimental data. Then, the fitting results $k = 183.7858$ and $n = 0.3870$ were used to define the viscosity in the software. The relative tolerance of 10^{-5} was set as the convergence criterion in solving continuity and momentum equations.

Table S1 Boundary settings of the CFD simulation

Boundaries	Setting
Inlet	Velocity inlet and the velocity is 0 m/s
Outlet	Pressure outlet and the gauge pressure is 0 Pa
Blade	Mobile wall and the velocity are the coating velocity
Wall	Stationary wall

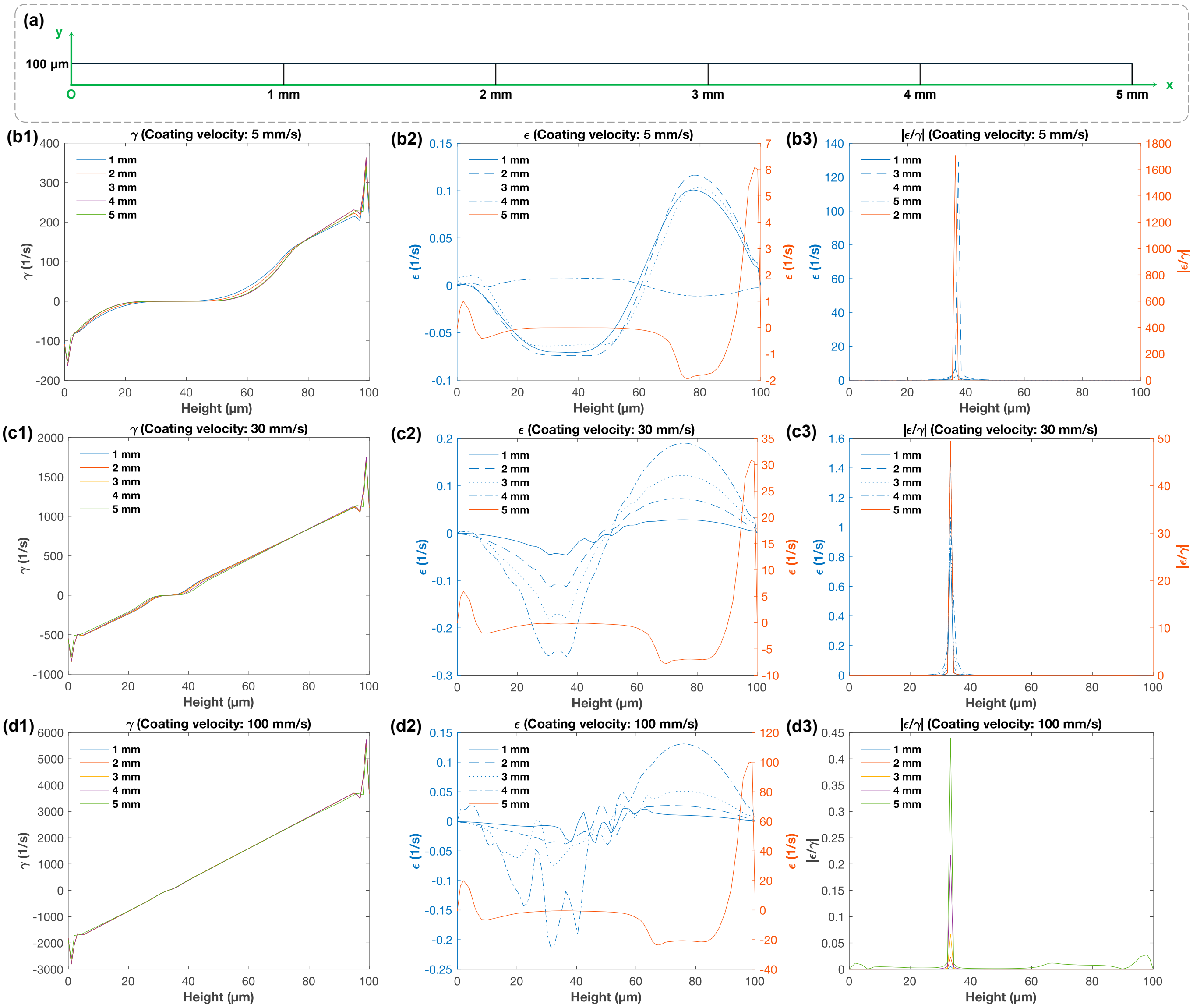

Figure S6 Detail of the simulation results. (**a**) The coordination used in the following illustrations. The shearing rate at different position when the coating velocity is (**b1**) 5mm/s, (**c1**) 30 mm/s, and (**d1**) 100 mm/s. The expansion rate at different position when the coating velocity is (**b2**) 5 mm/s, (**c2**) 30 mm/s, and (**d2**) 100 mm/s. The ratio of shearing rate and expansion rate at different position when the coating velocity is (**b3**) 5mm/s, (**c3**) 30 mm/s, and (**d3**) 100 mm/s.

Table S2 Thermal conductivity of all composites prepared at a coating thickness of 100 μm

Materials	$\lambda_{\parallel}$ (W/mK)	Deviation of $\lambda_{\parallel}$ (%)	$\lambda_{\perp}$ (W/mK)	Deviation of $\lambda_{\perp}$ (%)
PDMS	0.164	±5.42%	0.164	±5.42%
R-BP-10	0.251	±3.54%	0.251	±3.54%
V-BP-10-100	0.246	±0.12%	0.322	±0.12%
R-BP-20	0.463	±0.12%	0.463	±0.12%
V-BP-20-100	0.395	±0.12%	0.967	±1.03%
R-BP-30	0.814	±1.92%	0.814	±1.92%
V-BP-30-5	0.542	±1.67%	0.897	±1.01%

V-BP-30-30	0.532	$\pm$0.04%	1.001	$\pm$0.04%
V-BP-30-100	0.501	$\pm$0.04%	1.241	$\pm$0.73%

The sample names in the Table S2 are designated according to V-BP-x-y, where x and y represent the loading of BN (wt.%) and the coating velocity (mm/s). R-BP-x corresponds to the BN/PDMS composites with x wt.% loading of randomly aligned BN.

Table S3 Thermal conductivity of V-BP composites with a 30 wt.% loading of BN prepared at a coating velocity of 100 mm/s and different coating thicknesses ranging from 100-1000 μm

Materials	$\lambda_{\parallel}$ (W/mK)	Deviation of $\lambda_{\parallel}$ (%)	$\lambda_{\perp}$ (W/mK)	Deviation of $\lambda_{\perp}$ (%)
V-BP-30-100-100	0.501	$\pm$0.04%	1.241	$\pm$0.73%
V-BP-30-500-100	0.520	$\pm$0.18%	1.027	$\pm$0.32%
V-BP-30-1000-100	0.530	$\pm$0.95%	0.878	$\pm$0.68%

The sample names in the Table S3 are designated according to V-BP-x-y-z, where x, y, and z represent the loading of BN (wt.%), the coating thickness (μm), and the coating velocity (mm/s), respectively.

Estimated deviation of thermal conductivity

Thermal conductivity λ (W/mK) is calculated by $\lambda = a \times \rho \times C_p$, where a, ρ, and C_p are thermal diffusivity (m^2/s), solid density (kg/m^3), and specific heat capacity (J/(kg·K)), respectively. As a, ρ, and C_p are mutually independent, the deviation of λ can be estimated according to the Law of Propagation of Error as followed:

$$\frac{\Delta\lambda}{\lambda} = 100\% \times \sqrt{\left(\frac{\Delta a}{a}\right)^2 + \left(\frac{\Delta\rho}{\rho}\right)^2 + \left(\frac{\Delta C_p}{C_p}\right)^2}$$

where $\Delta\lambda$ is the estimated deviation of λ, Δa, $\Delta\rho$, ΔC_p are the standard deviation of a, ρ, and C_p, respectively.

Table S4 Thermal conductivity enhancement (TCE) of this work in comparison to other composites containing boron nitride reported by other studies

Reference	Composite	Fillers	Content of BN (wt.%)	TCE (%)
[18]	Epoxy/BN-PVDF	BN, PVDF	21	622
[37]	BN/epoxy	BN	17.57	204
[38]	2 wt% AgNW/20wt% BNNS/PDMS	AgNW, BNNS	20	237
[39]	PI/o-BNNS	BNNS	20	197
[40]	BN/NR	BN	25	339
[41]	BN/epoxy	BN	30	424
[42]	BN/LCER	BN	30	100
[43]	S-BN*/epoxy	BN	9.1	132
[44]	D-BN@PVB/EP	BN, PVB	40	305
This work	V-BP-30-100	BN	30	657

References

18. Chen, X.; Lim, J.S.K.; Yan, W.; Guo, F.; Liang, Y.N.; Chen, H.; Lambourne, A.; Hu, X. Salt Template Assisted BN Scaffold Fabrication toward Highly Thermally Conductive Epoxy Composites. *ACS Appl. Mater. Interfaces* **2020**, *12*, 16987–16996, doi:10.1021/acsami.0c04882.

37. Li, X.; Xu, Q.; Lei, Z.; Chen, Z. Electrostatic Flocking Assisted Aligned Boron Nitride Platelets Scaffold for Enhancing the Through-Plane Thermal Conductivity of Flexible Thermal Interface Materials. *Ceramics International* **2023**, *49*, 22623–22629, doi:10.1016/j.ceramint.2023.04.044.

38. Wang, S.; Li, W.; Jin, X.; Wu, J.; Chen, K.; Gan, W. Facile Fabrication of Three-Dimensional Thermal Conductive Composites with Synergistic Effect of Multidimensional Fillers. *J Mater Sci* **2021**, *56*, 12671–12685, doi:10.1007/s10853-021-06105-8.

39. Dong, J.; Cao, L.; Li, Y.; Wu, Z.; Teng, C. Largely Improved Thermal Conductivity of PI/BNNS Nanocomposites Obtained by Constructing a 3D BNNS Network and Filling It with AgNW as the Thermally Conductive Bridges. *Composites Science and Technology* **2020**, *196*, 108242, doi:10.1016/j.compscitech.2020.108242.

40. An, D.; Duan, X.; Cheng, S.; Zhang, Z.; Yang, B.; Lian, Q.; Li, J.; Sun, Z.; Liu, Y.; Wong, C.-P. Enhanced Thermal Conductivity of Natural Rubber Based Thermal Interfacial Materials by Constructing Covalent Bonds and Three-Dimensional Networks. *Composites Part A: Applied Science and Manufacturing* **2020**, *135*, 105928, doi:10.1016/j.compositesa.2020.105928.

41. Isarn, I.; Ferrando, F.; Serra, À.; Urbina, C. Novel BN-Epoxy/Anhydride Composites with Enhanced Thermal Conductivity. *Polymers for Advanced Technologies* **2021**, *32*, 1485–1492, doi:10.1002/pat.5184.

42. Yang, X.; Zhu, J.; Yang, D.; Zhang, J.; Guo, Y.; Zhong, X.; Kong, J.; Gu, J. High-Efficiency Improvement of Thermal Conductivities for Epoxy Composites from Synthesized Liquid Crystal Epoxy Followed by Doping BN Fillers. *Composites Part B: Engineering* **2020**, *185*, 107784, doi:10.1016/j.compositesb.2020.107784.

43. Yan, W.; Chen, X.; Lim, J.S.K.; Chen, H.; Gill, V.; Lambourne, A.; Hu, X. Epoxy-Assisted Ball Milling of Boron Nitride towards Thermally Conductive Impregnable Composites. *Composites Part A: Applied Science and Manufacturing* **2022**, *156*, 106868, doi:10.1016/j.compositesa.2022.106868.

44. Yang, F.; Sun, X.; Guo, Q.; Yao, Z. Improvement of Thermal Conductivities for Epoxy Composites via Incorporating Poly(Vinyl Benzal)-Coated h-BN Fillers and Solvent-Assisted Dispersion. *Ind. Eng. Chem. Res.* **2019**, *58*, 18635–18643, doi:10.1021/acs.iecr.9b03861.
